# Human antigen R regulates hypoxia‐induced mitophagy in renal tubular cells through PARKIN/BNIP3L expressions

**DOI:** 10.1111/jcmm.16301

**Published:** 2021-01-26

**Authors:** Shao‐Hua Yu, Kalaiselvi Palanisamy, Kuo‐Ting Sun, Xin Li, Yao‐Ming Wang, Feng‐Yen Lin, Kuen‐Bao Chen, I‐Kuan Wang, Tung‐Min Yu, Chi‐Yuan Li

**Affiliations:** ^1^ Graduate Institute of Biomedical Sciences China Medical University Taichung Taiwan; ^2^ Department of Emergency Medicine China Medical University Hospital Taichung Taiwan; ^3^ Department of Pediatric Dentistry China Medical University Hospital Taichung Taiwan; ^4^ School of Dentistry, College of Dentistry China Medical University Taichung Taiwan; ^5^ Department of Radiology Taichung Tzu Chi Hospital Buddhist Tzu Chi Medical Foundation Taichung Taiwan; ^6^ Department of Internal Medicine School of Medicine College of Medicine Taipei Medical University Taipei Taiwan; ^7^ Division of Cardiology and Cardiovascular Research Center Taipei Medical University Hospital Taipei Taiwan; ^8^ School of Medicine China Medical University Taichung Taiwan; ^9^ Department of Anesthesiology China Medical University Hospital Taichung Taiwan; ^10^ Division of Nephrology China Medical University Hospital Taichung Taiwan; ^11^ Division of Nephrology Department of Internal Medicine Taichung Veterans General Hospital Taichung Taiwan

**Keywords:** acute kidney injury, BNIP3L, HK‐2, HuR, mitophagy, PARKIN

## Abstract

Mitochondrial dysfunction contributes to the pathophysiology of acute kidney injury (AKI). Mitophagy selectively degrades damaged mitochondria and thereby regulates cellular homeostasis. RNA‐binding proteins (RBPs) regulate RNA processing at multiple levels and thereby control cellular function. In this study, we aimed to understand the role of human antigen R (HuR) in hypoxia‐induced mitophagy process in the renal tubular cells. Mitophagy marker expressions (PARKIN, p‐PARKIN, PINK1, BNIP3L, BNIP3, LC3) were determined by western blot analysis. Immunofluorescence studies were performed to analyze mitophagosome, mitolysosome, co‐localization of p‐PARKIN/TOMM20 and BNIP3L/TOMM20. HuR‐mediated regulation of PARKIN/BNIP3L expressions was determined by RNA‐immunoprecipitation analysis and RNA stability experiments. Hypoxia induced mitochondrial dysfunction by increased ROS, decline in membrane potential and activated mitophagy through up‐regulated PARKIN, PINK1, BNIP3 and BNIP3L expressions. HuR knockdown studies revealed that HuR regulates hypoxia‐induced mitophagosome and mitolysosome formation. HuR was significantly bound to *PARKIN* and *BNIP3L* mRNA under hypoxia and thereby up‐regulated their expressions through mRNA stability. Altogether, our data highlight the importance of HuR in mitophagy regulation through up‐regulating PARKIN/BNIP3L expressions in renal tubular cells.

## 1 INTRODUCTION

Mitochondria provide high energy needs to perform pleiotropic functions of the kidney such as metabolism, nutrients' reabsorption, fluid and electrolytes' balance.^1^ Mitochondrial dysfunction caused by ischemia‐reperfusion injury is one of the most important contributor in AKI pathogenesis.^2^ Maintenance of mitochondrial health is pivotal as they are prime source of ROS and apoptotic regulators. ^3^ Dysfunction causes decreased ATP production and activation of mitochondrial stress responses. Loss of mitochondrial function has been previously reported in experimental AKI models of sepsis and cisplatin‐induced nephrotoxicity.^4^, ^5^, ^6^, ^7^ Disturbed mitochondrial dynamics, redox status and energetics are all implicated during AKI.^5^, ^8^, ^9^, ^10^ Thus, mitochondria regulate complex cellular signalling of cell survival and death mechanisms.^11^ Removal of dysfunctional mitochondria by organelle‐specific autophagy termed as mitochondrial autophagy (mitophagy) replenishes with functional mitochondria.^12^ Impaired mitophagy mechanisms might promote inflammation and cell death causing AKI progression to chronic kidney disease (CKD).^13^ Since mitophagy process maintains functional mitochondria it is important to understand its regulatory mechanisms.

Mitophagy is a protective response against oxidative damage in AKI.^14^ During increased oxidative stress, dysfunctional mitochondria with relatively low membrane potential are segregated during mitochondrial fission process.^15^ Further, priming of mitochondria through PINK1 (PTEN‐induced kinase 1)/PARKIN (Parkin RBR E3 ubiquitin protein ligase) and BNIP3 (BCL2 interacting protein 3)/BNIP3L (BCL2 interacting protein 3 like)‐dependent mechanisms help in recognition by autophagic machinery to form mitophagosome. Finally, mitochondria degradation occurs in the endolysosomal compartment (mitolysosme) through the fusion of mitophagosome with lysosomes. Studies show the functional role of mitophagy in the pathogenesis of disease.^16^, ^17^ Renoprotective role of mitophagy has been demonstrated in a hyperglycaemic rabbit model,^18^ acid‐loaded metabolic acidosis,^19^ high‐calorie diet‐induced injury,^20^ ischemia‐reperfusion injury,^17^ sepsis^21^ and kidney fibrosis.^22^ Despite abundant knowledge on mitophagy, the regulatory mechanisms involved in the removal of dysfunctional mitochondria remains elusive. For a better therapeutic strategy, understanding the homeostatic regulatory mitophagy mechanisms involved in AKI is paramount.

RNA‐binding proteins (RBPs) regulate gene expression through post‐transcriptional processing of RNA. RBPs regulate cellular adaptation to stress response by modulating functionally related proteins through RNA regulation.^23^ RBPs specifically bind to the A/U rich regions of the target transcripts through RNA‐binding domains (RBDs) and regulate its mRNA stability and translation.^24^ In addition, these RBPs bound to the AU rich regions at 3'UTRs of RNA transcripts protect from miRNA‐mediated translational repression.^25^, ^26^, ^27^ HuR is ubiquitously expressed RBP which is mainly involved in post‐transcriptional regulation of RNA. HuR belongs to embryonic lethal abnormal vision (ELAV) family of Hu proteins and regulates cellular functions including proliferation, immune regulation, differentiation, senescence, apoptosis and stress responses.^28^ The functional importance of RNA‐binding proteins in regulating nuclear‐encoded mitochondrial protein expression and mitochondrial function has been reviewed elsewhere.^29^ We have previously demonstrated that under hypoxia, HuR regulates cellular autophagy and apoptosis in renal cells.^30^ However, the importance of RBPs in mitophagy process is not clear. In this study, we focussed on identifying whether HuR might play an important regulatory role in mitophagy.

Our findings show novel evidence that HuR functionally regulates mitophagy under hypoxia‐induced stress in renal tubular cells. HuR‐mediated post‐transcriptional function up‐regulates PARKIN and BNIP3L expressions through mRNA stabilization and thereby regulates hypoxia‐induced mitophagy in renal tubular cells.

## 2 MATERIALS AND METHODS

### 2.1 Cells, antibodies and reagents

HK‐2 cells (Human, kidney proximal tubular 2) were obtained from ATCC (ATCC^®^ CRL‐2190™). The primary antibodies used in this study include anti‐PARKIN (Cat no: #2132, 1:500; Cell Signaling Technology), anti‐p‐PARKIN (Cat no: orb312554, 1:250, Biorbyt), anti‐PINK1 (Cat no: #6946, 1:500; Cell Signaling Technology), anti‐BNIP3L (Cat no: GTX111876, 1:1000, GeneTex), anti‐BNIP3 (Cat no: GTX10433, 1:1000, GeneTex), anti‐HuR (Cat no: #12582, 1:1000, Cell Signaling Technology), anti‐TOMM20 (Cat no: ab56783, 1:200, Abcam), anti‐LC3 (Cat no: NB100‐2220, 1:200, Novus Biologicals), anti‐COXIV (Cat no: 4850, 1:1000, Cell Signaling Technology), anti‐LAMP2 (Cat no: 49067, 1:200, Cell Signaling Technology), and anti‐β‐ACTIN (Cat no: A1978, 1:1000, Sigma‐Aldrich). Secondary antibodies‐rabbit/mouse IgG HRP, Alexa Fluor 546 anti‐mouse IgG (H + L), Alexa Fluor 488 anti‐goat IgG (H + L), Alexa Fluor 647 anti‐rabbit IgG (H + L) were purchased from Invitrogen™. 2′,7′‐dichlorofluorescin‐diacetate (DCFH2‐DA), JC‐1 and DAPI (4′,6‐diamidino‐2‐phenylindole) mount were purchased from Sigma Aldrich. Magna RIP™ RNA‐Binding Protein Immunoprecipitation Kit was procured from Sigma‐Aldrich. The SuperSignal West Femto Maximum Sensitivity Substrate detection reagents were purchased from Thermo Scientific Inc.

### 2.2 Cell culture and treatment

HK‐2 cells were cultured in RPMI 1640 medium with 10% FBS and 1% penicillin/streptomycin in a CO_2_ incubator at 37°C. The cells were treated under normoxia or 1% hypoxic conditions at indicated time points and harvested for further analysis.

### 2.3 Generation of HuR‐knockdown cells

Lentivirus‐mediated stable HK‐2‐shHuR knockdown cells were established as described previously.^30^ The HuR and LacZ‐control shRNA targeting sequences in pLKO.1‐puro vector and packaging plasmids (pMD.G, pCMVDR8.91) were purchased from RNAiCore facility, Academia Sinica, Taiwan.

### 2.4 Confocal imaging

The HK‐2 cells or HuR knockdown cells were allowed to attach in coverslips and then exposed to normoxia or hypoxia conditions. Followed by fixation, the cells were permeabilized with Triton X‐100‐blocking buffer for 60 minutes. The cells were treated with primary antibodies overnight at 4°C. HuR localization in the nucleus/cytoplasm under normoxia and hypoxia was determined using anti‐HuR primary antibody and DAPI counter staining. To examine p‐PARKIN or BNIP3L mitochondrial localization, the cells were stained with anti‐p‐PARKIN or anti‐BNIP3L primary antibodies and co‐incubated with anti‐TOMM20 (Translocase of outer mitochondrial membrane 20) antibody (mitochondrial marker). To monitor hypoxia‐induced mitophagosome formation, the cells were incubated with anti‐LC3 (microtubule associated protein 1 light chain 3 alpha) and anti‐TOMM20 primary antibodies and mitolysosome formation were assessed through primary antibodies specific to LAMP2 (lysosomal associated membrane protein 2) (lysosome marker) and COXIV (cytochrome c oxidase subunit 4I1) (mitochondrial marker). The coverslips were washed with PBS for 3 times, 5 minutes each. The cells were incubated with a secondary antibody (1:100) for 2 hours at room temperature. The cells were DAPI mounted and analysed under Olympus DSU Spinning Disk‐confocal microscope. Pearson's coefficient for co‐localization of LC3/TOMM20, LAMP2/COXIV, p‐PARKIN/TOMM20, BNIP3L/TOMM20 were analysed using the JACoP plugin, ImageJ software.

### 2.5 RNA purification and RT‐qPCR

The total RNA isolated (TRIzol reagent) was reverse transcribed to cDNA using M‐MLV Reverse Transcriptase. Quantitative real‐time PCR analysis for *PARKIN* and *BNIP3L* expressions was performed using SYBR green fluorescent mix (Thermo Fisher Scientific). *GAPDH* was used as a normalization control. Primers specific for *PARKIN* (Forward‐ GGAGCTGAGGAATGACTGGA; Reverse: ACAATGTGAACAATGCTCTGCT), *BNIP3L* (Forward‐ GATGCACAACATGAATCAGGA; Reverse: CCATCTTCTTGTGGCGAAG), GAPDH (Forward‐AGCCACATCGCTCAGACAC; Reverse: GCCCAATACGACCAAATCC) were used.

### 2.6 Mitochondrial superoxide quantification

The mitochondrial superoxide levels were detected using mitochondrial superoxide detection kit (Abcam 19943). The HK‐2 cells were allowed for overnight attachment and exposed for normoxia and hypoxia (6 and 16 hours) conditions. Followed by this, mitoROS working solution was added and incubated at 37℃ for 60 minutes. The fluorescence intensity was measured at 540/590 nm using fluorescence reader (Hitachi Spectrofluorometer).

### 2.7 Mitochondrial membrane potential (Δψm)

The mitochondrial membrane potential assay was performed using JC‐1 cell‐permeant dye. After the respective treatment schedule of normoxia and hypoxia, cells were treated with JC‐1 dye for 30 minutes. The cells were PBS washed, and fluorescence intensity was measured using a fluorescence reader (Hitachi Spectrofluorometer).

### 2.8 Western blotting

The cells were exposed to normoxia or hypoxia, and the proteins were isolated using RIPA buffer. The SDS‐PAGE gel (10%‐12%) separated proteins were transferred to PVDF membrane (PerkinElmer, Life Science). The membranes were incubated in the blocking buffer for 1 minute and treated with primary antibodies overnight at 4°C. The blots were washed with TBST (thrice, 5 minutes each) and incubated with IgG HRP‐linked secondary antibodies for 1 hour at room temperature. The membranes were washed with TBST and exposed to enhanced chemiluminescence (ECL) solution for 1‐2 minutes, and images were captured in ImageQuant LAS4000 (GE Healthcare).

### 2.9 HuR‐RNA immunoprecipitation

The RNA immunoprecipitation was performed to analyse HuR bound *PARKIN* and *BNIP3L* mRNAs. The experiment was performed as per the manufacturer's protocol (Magna RIP™ RNA‐Binding Protein Immunoprecipitation Kit; Millipore). The HuR‐RNA complexes in the cell lysate were immunoprecipitated using protein G beads‐HuR antibody. The purified RNA was analysed for *PARKIN* and *BNIP3L* expressions using RT‐qPCR analysis.

### 2.10 mRNA stability

To determine HuR‐mediated RNA stability, WT and knockdown HuR cells were treated with actinomycin D (2.5 μg/mL). The isolated RNAs after treatment were analysed for *PARKIN* and *BNIP3L* expressions using RT‐qPCR analysis and half‐life was calculated. *7SL*, stable lncRNA was used as a positive control.

### 2.11 Statistics

Data are expressed as mean values ± SD. The significant differences were calculated using one‐way or two‐way analysis of variance followed by Dunnett's or Sidak's or Tukey's multiple comparison analysis. The differences were considered statistically significant for *P* < .05, *P* < .01, *P* < .001. NS represents non‐significant. The data were analysed using Prism6 (GraphPad Software Inc).

## 3 RESULTS

### 3.1 Hypoxia induces mitochondrial dysfunction and mitophagy proteins (PINK1/PARKIN, BNIP3/BNIP3L) in renal tubular cells

**FIGURE 1:**
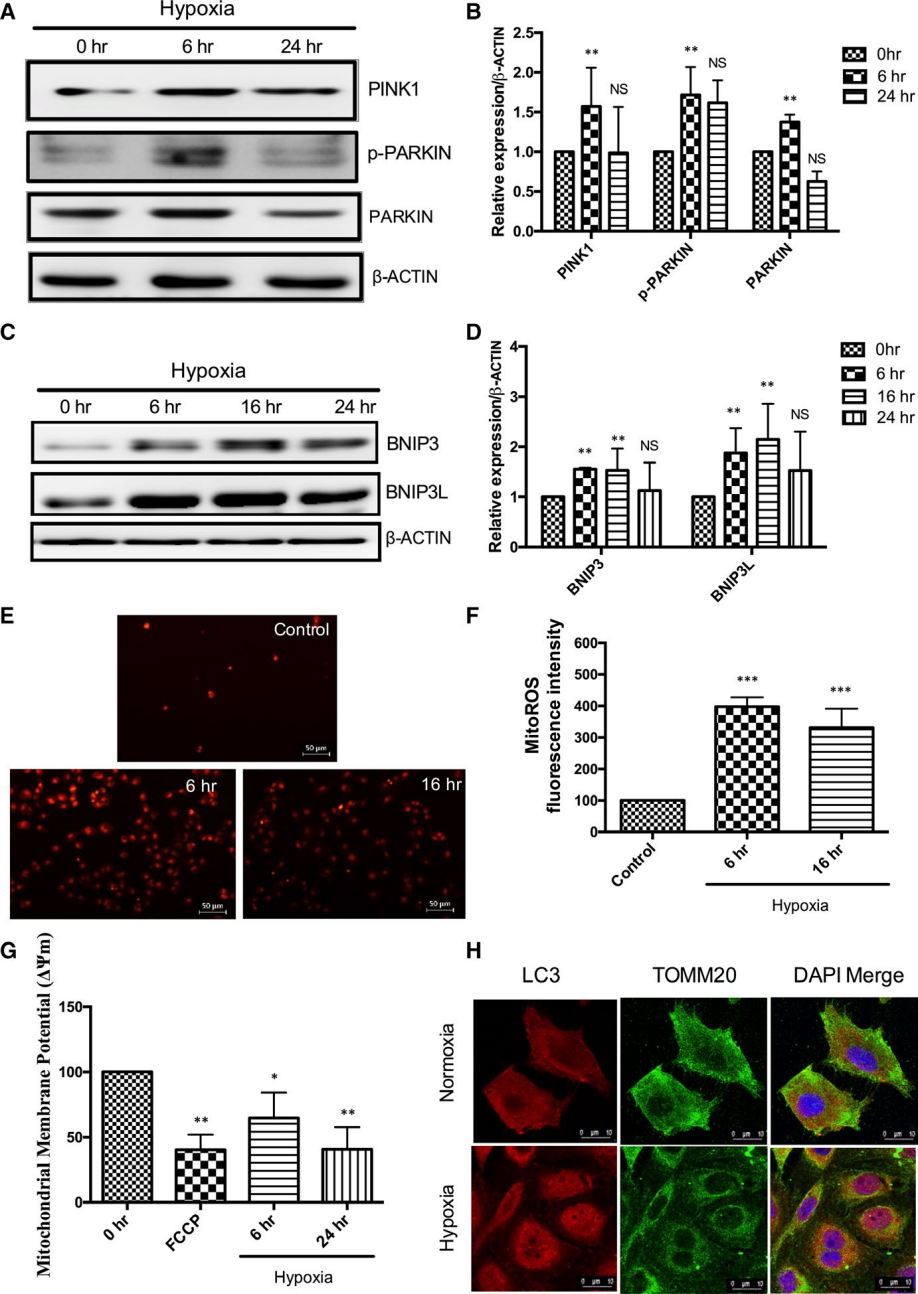
Hypoxia induces mitochondrial dysfunction and mitophagy in HK‐2 cells. (A, B) Hypoxia induces PINK1, p‐PARKIN and PARKIN expression under hypoxia. Results of densitometric analysis shown as mean ± SD, n = 3; non‐significant (NS),**P < .01 compared with 0 hour, two‐way ANOVA followed by Tukey's multiple comparisons test. (C, D) Increased BNIP3 and BNIP3L expressions under hypoxia. Results of densitometric analysis shown as mean ± SD, n = 3; non‐significant (NS),**P < .01 compared with 0 hour, two‐way ANOVA followed by Dunnett's multiple comparisons test. (E, F) Hypoxia induces MitoROS fluorescence intensity, Scale‐50 µm, Magnification‐200×; (G) Hypoxia induces change in mitochondrial membrane potential. The results are shown as mean ± SD, n = 3, *P < .05,**P < .01,***P < .001 compared with control, one‐way ANOVA followed by Dunnett's multiple comparisons test. (H) Hypoxia induces LC3 localization with mitochondria (TOMM20). LC3‐Red; TOMM20 (green); DAPI (Blue). Scale‐10 µm, Magnification‐1,260×

In order to understand the effect of hypoxia stress on mitochondrial dysfunction, we analysed mitochondrial ROS, mitochondrial membrane potential and mitophagy‐related protein expressions in the renal tubular cells. Figure 1A,B shows significant up‐regulation of PINK1, PARKIN, p‐PARKIN expressions in HK‐2 cells at 6 hours hypoxia compared to 0 hour. Figure 1C,D shows a significant increase in BNIP3, BNIP3L expressions under 6 hours, 16 hours hypoxia compared to 0 hour. Further, hypoxia induced oxidative stress in renal cells by increasing mitochondrial superoxide levels with concomitant mitochondrial depolarization (Figure 1E‐G). Since mitophagy protein expressions were significantly up‐regulated at 6 hours hypoxia, further experiments were carried out at this time point. Figure 1H shows that hypoxia increased LC3 mitochondrial translocation under hypoxia leading to mitophagosome formation compared to control cells. These results show that hypoxia induces mitophagy through increased mitophagosome formation in HK‐2 cells.

### 3.2 Human antigen R (HuR) regulates hypoxia‐induced mitophagosome formation in renal tubular cells

**FIGURE 2:**
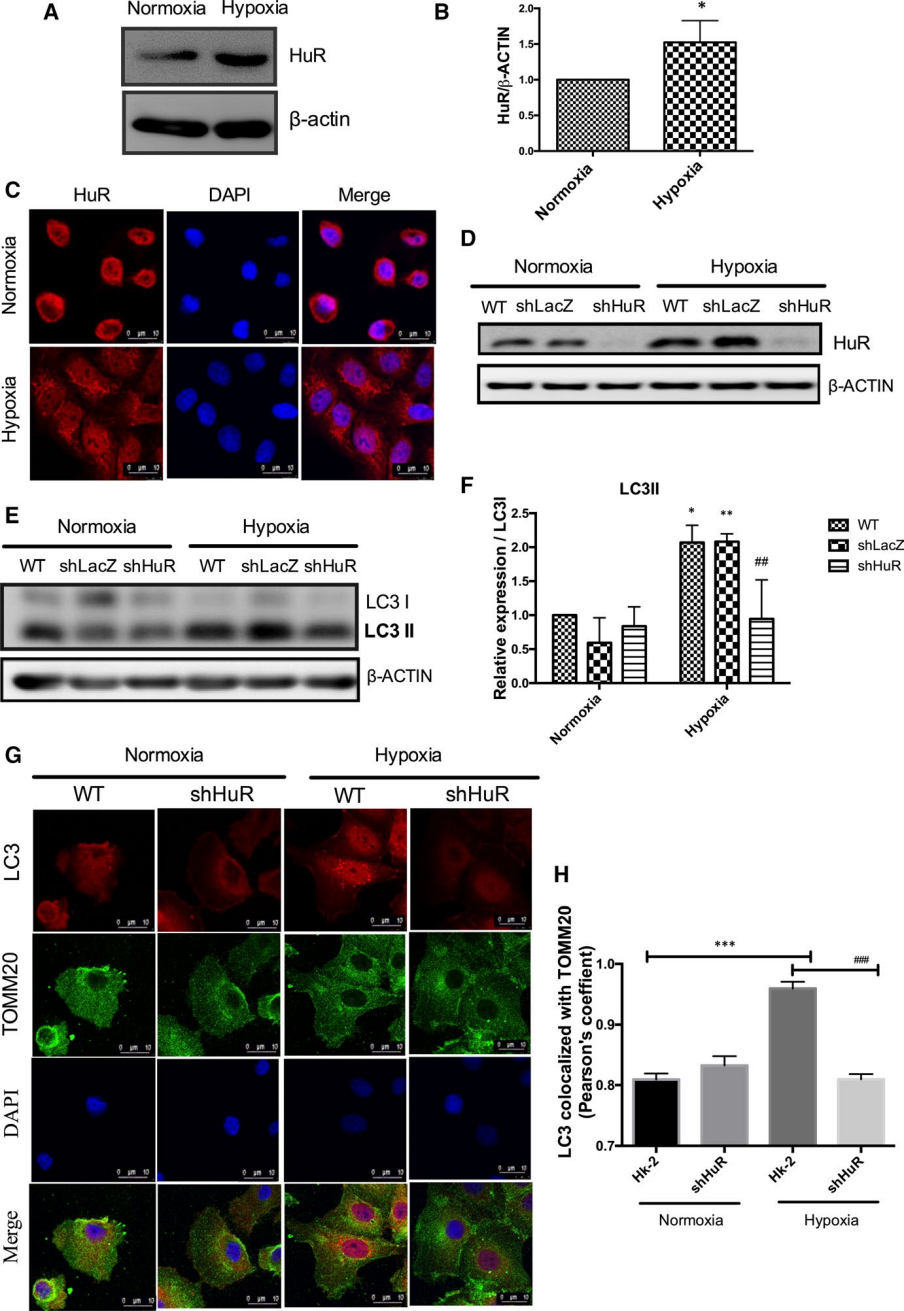
HuR regulates hypoxia‐induced mitophagosome formation in renal tubular cells. (A, B) Hypoxia up‐regulates HuR expression compared to normoxia. The data are expressed as mean ± SD, n = 3, two‐tailed unpaired *t*‐test, *P < .05 compared to normoxia. (C) Confocal analysis of cytoplasmic localization of HuR under hypoxia. Red‐HuR, Blue‐DAPI. Scale‐10 µm, Magnification‐1260× (D) HK‐2 shHuR cells shows down‐regulated HuR expression under normoxia and hypoxia. (E, F) Knockdown HuR significantly down‐regulates hypoxia‐induced LC3II expression. The relative density of LC3II/LC3I expression is represented as mean ± SD, n = 3, *P < .05, **P < .01, compared with WT‐normoxia; ^##^ *P* < .01 compared with WT‐hypoxia. Two‐way ANOVA followed by Sidak's multiple comparisons test. (G) Effect of HuR on mitophagosome formation under hypoxia as analysed by immunofluorescence analysis. LC3‐Red; TOMM20 (green); DAPI (Blue). Scale‐10 µm, Magnification‐1,260× (H) The bar graph shows Pearson's coefficient for the co‐localization of LC3 with TOMM20. The values are mean ± SD, n = 3, ***P < .001 compared with HK‐2 normoxia, ^###^ *P* < .001 compared with HK‐2‐Hypoxia, two‐way ANOVA followed by Dunnett's multiple comparisons test

To identify whether the RNA‐binding protein (HuR) regulates hypoxia‐induced mitophagy, the HuR expression was analysed under normoxia and hypoxia. Figure 2A,B shows that hypoxia up‐regulates HuR expression compared to normoxia. Further, HuR translocated from nucleus to cytoplasm under hypoxia (Figure 2C). To evaluate the role of HuR on mitophagosome formation, we established knockdown HuR (shHuR) cells (Figure 2D). Hypoxia‐induced LC3II expressions was significantly decreased in the HuR knockdown cells (Figure 2E,F). Immunofluorescence results of LC3 co‐localization with TOMM20 show that HuR knockdown cells significantly reduced hypoxic stress‐induced mitophagosome formation (Figure 2G,H).

### 3.3 HuR regulates mitochondria co‐localization with lysosomes

**FIGURE 3:**
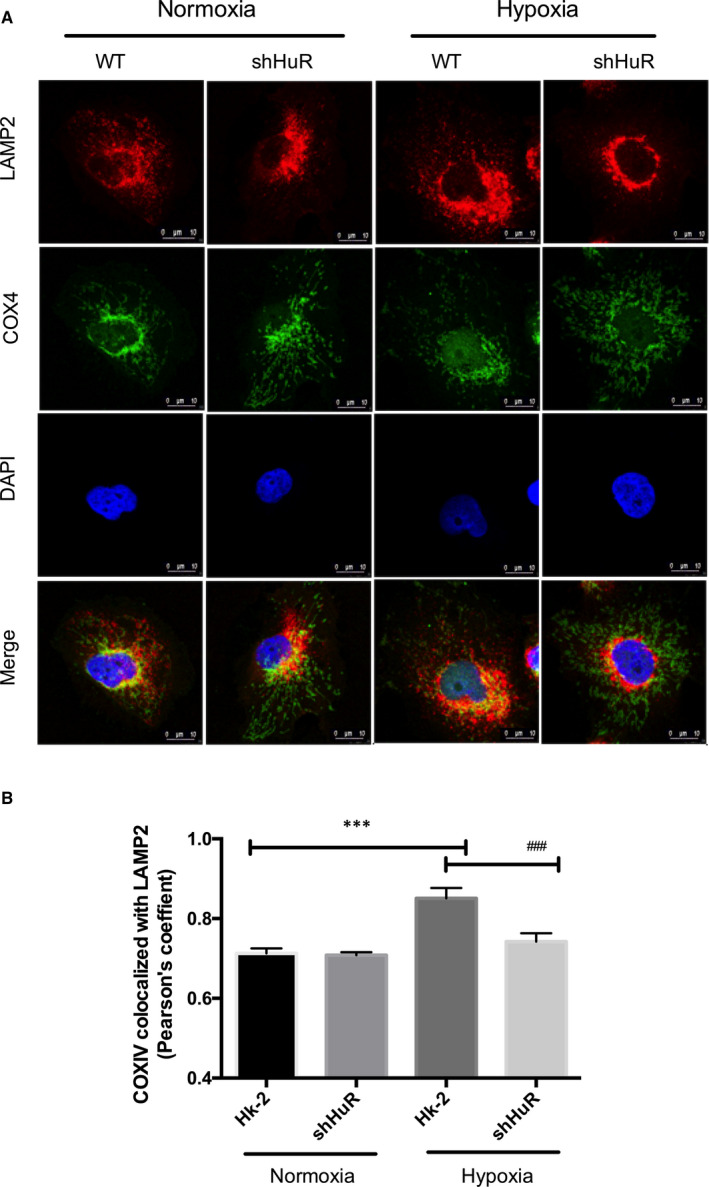
HuR regulates mitochondria co‐localization with lysosomes under hypoxia. (A) The effect of HuR on hypoxia‐induced mitolysosome in HK‐2 and shHuR cells. Immunofluorescence results showing HuR regulates LAMP2 (lysosome) co‐localization with COXIV (mitochondria). LAMP2‐Red; COXIV (green); DAPI (Blue). Scale‐10 µm, Magnification‐1,260× (B) The bar graph shows Pearson's coefficient for the co‐localization of COXIV with LAMP2. The values are mean ± SD, n = 3, ***P < .001 compared with HK‐2 normoxia, ^###^ *P* < .001 compared with HK‐2‐Hypoxia, two‐way ANOVA followed by Dunnett's multiple comparisons test

Further, we analysed the involvement of HuR on mitolysosome formation under hypoxia. Figure 3A,B shows that HuR regulated hypoxia‐induced mitochondria (COX1V) co‐localization with lysosomes (LAMP2) by immunofluorescence studies. The findings show that, HuR regulated mitolysosome formation under hypoxia in renal tubular cells.

### 3.4 HuR regulates hypoxia‐induced PARKIN expression

**FIGURE 4:**
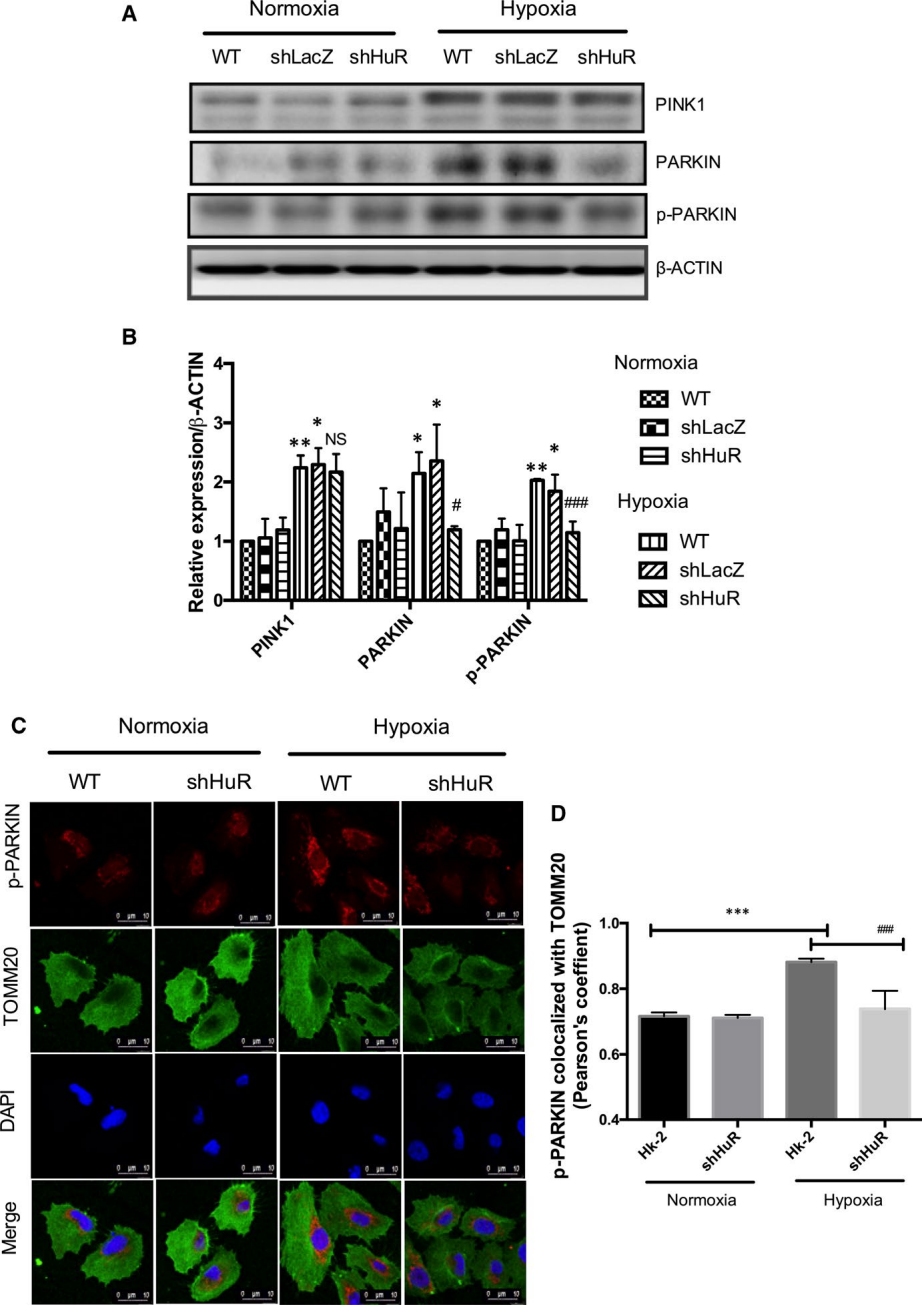
HuR regulates hypoxia‐induced p‐PARKIN/PARKIN expression. (A, B) Effect of HuR on hypoxia‐mediated mitophagy protein (PINK1, p‐PARKIN and PARKIN) expressions. The relative density of western blot results shows that shHuR cells significantly down‐regulated p‐PARKIN and PARKIN expressions compared to WT cells under hypoxia. The results are shown as mean ± SD, n = 3, *P < .05, **P < .01 compared to WT‐normoxia; non‐significant (NS), ^#^ *P* < .05, ^###^ *P* < .001, compared to WT‐hypoxia; two‐way ANOVA followed by Sidak's multiple comparisons test. (C) Immunofluorescence showing p‐PARKIN translocation to mitochondria (TOMM20) regulated by HuR under hypoxia. p‐PARKIN‐Red; TOMM20 (green); DAPI (Blue). Scale‐10 µm, Magnification‐1260X (D) Pearson's coefficient for the co‐localization of p‐PARKIN with TOMM20. The values are mean ± SD, n = 3, ***P < .001 compared with HK‐2 normoxia, ^###^ *P* < .001 compared with HK‐2‐Hypoxia, two‐way ANOVA followed by Dunnett's multiple comparisons test

PARKIN/PINK1 mediates mitophagy process and removes dysfunctional mitochondria.^16^ We analysed whether HuR regulates PARKIN/PINK1‐mediated mitophagy in renal tubular cells. Figure 4A,B shows that hypoxia up‐regulated PARKIN, p‐PARKIN, PINK1 expressions under hypoxia compared to control cells. However, knockdown HuR cells significantly down‐regulated the PARKIN and p‐PARKIN expressions under hypoxia. The immunofluorescence studies show that hypoxia increases mitochondrial localization of p‐PARKIN under hypoxia compared to normoxia. Further, there was significant reduction of mitochondrial p‐PARKIN in HuR knockdown cells compared to WT cells under hypoxia (Figure 4C,D). The results show that HuR regulates PARKIN expression during hypoxia‐induced mitophagy in HK‐2 cells.

### 3.5 HuR regulates BNIP3L expression under hypoxia

**FIGURE 5:**
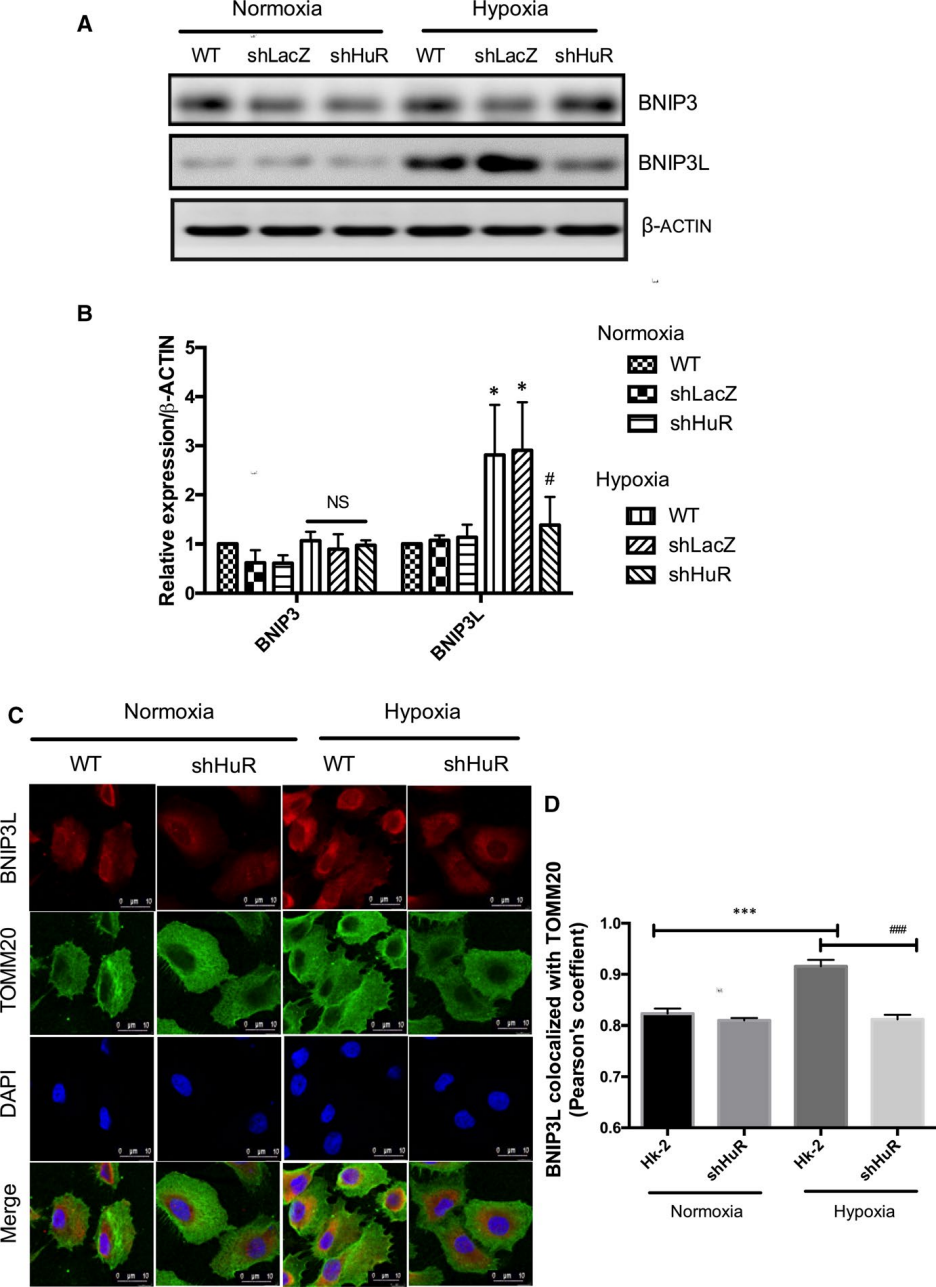
HuR regulates hypoxia‐induced BNIP3L expression. (A, B) Effect of HuR on hypoxia‐induced BNIP3 and BNIP3L expressions. The relative density of western blot results shows that shHuR cells significantly down‐regulated BNIP3L expressions compared to control cells under hypoxia. The results are shown as mean ± SD, n = 3, *P < .05, compared to WT‐normoxia; non‐significant (NS), ^#^ *P* < .05, compared to WT‐hypoxia; two‐way ANOVA followed by Sidak's multiple comparisons test. (C) Immunofluorescence studies on BNIP3L translocation to mitochondria (TOMM20) regulated by HuR under hypoxia. BNIP3L‐Red; TOMM20 (green); DAPI (Blue). Scale‐10 µm, Magnification‐1260X (D) Pearson's coefficient for the BNIP3L co‐localization with TOMM20. The values are mean ± SD, n = 3, ***P < .001 compared with HK‐2 normoxia, ^###^ *P* < .001 compared with HK‐2‐Hypoxia, two‐way ANOVA followed by Dunnett's multiple comparisons test

In order to assess whether HuR regulates BNIP3/BNIP3L pathway of mitophagy, we analysed their expressions in WT and shHuR cells under hypoxia. The results show that hypoxia up‐regulated BNIP3L expressions were significantly down‐regulated in HuR knockdown cells. However, HuR deficiency did not down‐regulate BNIP3 expressions under hypoxia (Figure 5A,B). To determine whether BNIP3L translocates to mitochondria under hypoxia, co‐localization studies were performed in WT and shHuR cells. The immunofluorescence results showed that knockdown HuR significantly reduced BNIP3L co‐localization with the mitochondria under hypoxia (Figure 5C,D). The findings reveal that HuR involves in the mitophagy process by regulating BNIP3L expression.

### 3.6 HuR promotes PARKIN/BNIP3L expressions through mRNA stability

**FIGURE 6:**
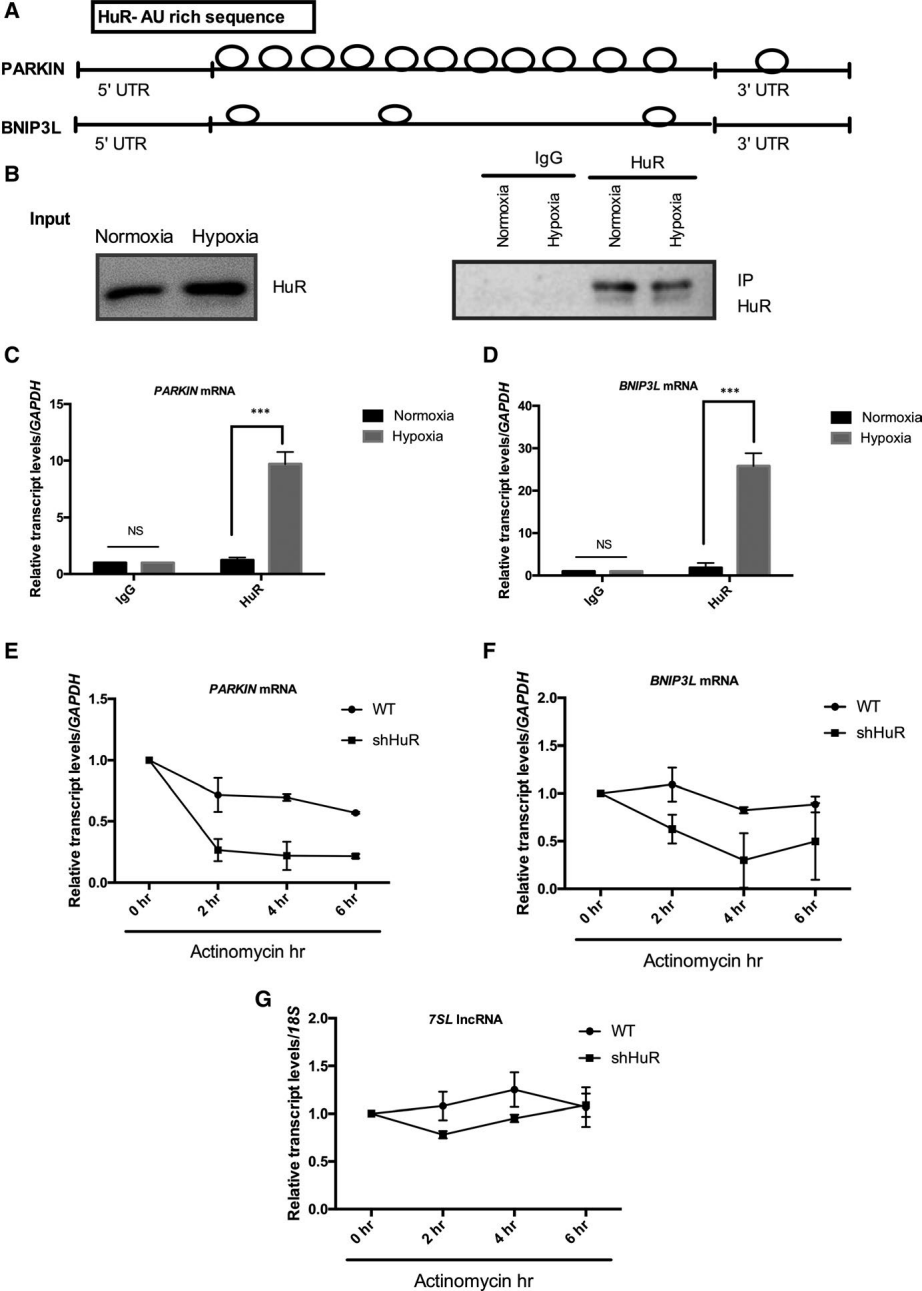
HuR regulates PARKIN and BNIP3L expressions through RNA stability. (A) Bioinformatics analysis showing HuR bound to A/U rich regions in *PARKIN* and *BNIP3L* mRNA. (B‐D) HuR RNA immunoprecipitation analysis shows *PARKIN* and *BNIP3L* mRNA were significantly bound to HuR under hypoxia. The results are shown as mean ± SD, n = 3, non‐significant (NS) between IgG under normoxia and hypoxia, ***P < .001 compared to normoxia; two‐way ANOVA followed by Sidak's multiple comparisons test. (E‐G) Effects of HuR on the mRNA stability of *PARKIN* and *BNIP3L* mRNA expressions were determined in the presence of actinomycin D. Stable lncRNA *7SL* was used as control

Further, to understand the mechanism of HuR‐mediated PARKIN and BNIP3L expressions, bioinformatics analysis was performed. From the AU‐Rich Element (ARE) database (https://brp.kfshrc.edu.sa/ared/Home/BasicSearch), we found that HuR binds to ARE sites on *PARKIN* and *BNIP3L* mRNA (Figure 6A). HuR bound RNAs were isolated by HuR‐IP and the results showed that HuR was significantly bound to *PARKIN* and *BNIP3L* mRNA under hypoxia conditions. (Figure 6 B‐D). To validate the effect of HuR, RNA stability experiments were performed to determine the mRNA half‐life of *PARKIN* and *BNIP3L*. Figure 6 E‐G, shows that the absence of HuR under hypoxic conditions declined the mRNA half‐life (t1/2) of *PARKIN* from 6 hours to <2 hours as compared to wild‐type HK‐2 cells. Similarly, HuR knockdown reduced *BNIP3L* half‐life (t1/2) from 6 hours to 3 hours. *7SL* lncRNA, a stable lncRNA was used as a positive control. Altogether, the results show that HuR binds and stabilizes *PARKIN* and *BNIP3L* mRNA and regulates its expression under hypoxia and in the renal tubular cells.

## 4 DISCUSSION

**FIGURE 7:**
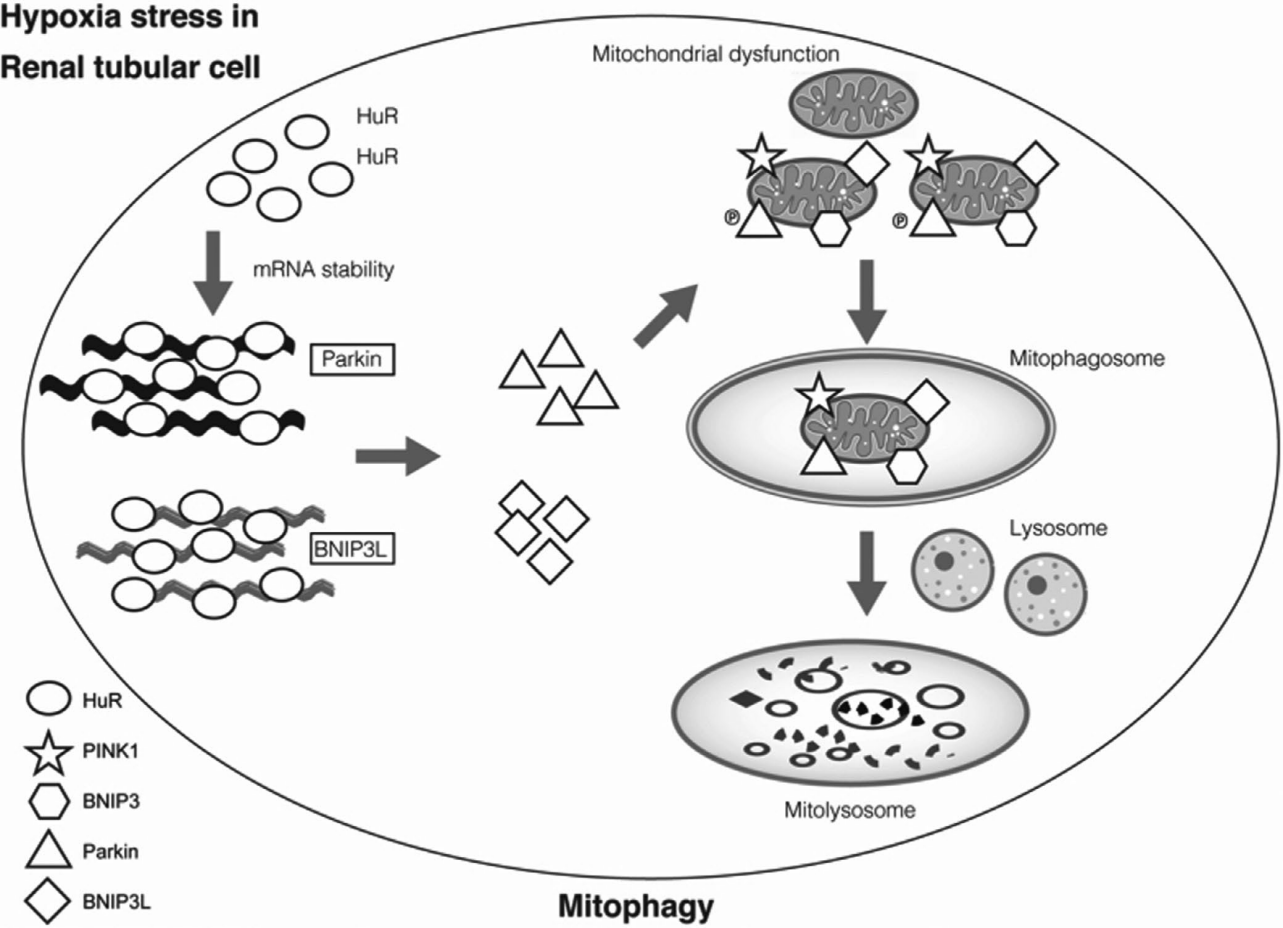
Mitophagy regulation by RNA‐Binding Protein‐HuR in HK‐2 cells

The findings of the study show that hypoxia induces mitophagy in the renal tubular cells. Further, the RNA‐binding protein (HuR) regulates PARKIN and BNIP3L expressions through post‐transcriptional stabilization of mRNA (Figure 7).

Mitochondria homeostasis plays a critical role in the pathogenesis of AKI. Mitochondria under stress undergo changes in mitochondrial membrane potential, mitochondrial biogenesis, mitochondrial fusion‐fission mechanisms and mitophagy.^31^ Elevated ROS levels and mitochondrial dysfunction has been reported in cisplatin‐induced I/R injury^32^ and sepsis‐associated AKI.^33^ Mitochondrial ROS generation and activation of mitophagy have been demonstrated in contrast‐induced AKI.^34^ The protective role of mitochondrial clearance through mitophagy has been demonstrated in kidney I/R injury.^35^ In addition, several studies demonstrate the adverse effects of impaired mitophagy.^36^, ^37^ Notably, insufficient mitochondrial autophagy in the hyperglycaemic rats showed severe organ failure of the liver and kidney.^38^ In this present study, we show a rise in oxidative stress was accompanied with up‐regulated mitophagy proteins (PARKIN, p‐PARKIN, PINK1, BNIP3 and BNIP3L) and mitophagosome formation under hypoxia in HK‐2 cells. Thus, hypoxia‐induced mitophagy might regulate mitochondrial function in renal tubular cells.

RNA‐binding proteins and its post‐transcriptional mechanisms maintain gene regulatory networks during normal physiology and pathological conditions.^39^ RBPs play a crucial role during adaptive cellular responses through mitochondrial functional reprogramming.^40^ RBPs are master regulators of translation efficiency and thereby maintain mitochondrial health and homeostasis. Several RBPs, including ZC3H10, SMG, LARRP, TTP, CLUH regulate oxidative phosphorylation.^41^, ^42^, ^43^, ^44^ Lin28a through its cold‐shock domain and CCHC‐type zinc fingers stimulates the translation of mitochondrial metabolic enzymes.^45^ The previous study on endoplasmic reticulum (ER) induced stress showed the predominant role of HuR and TIA‐1 on the regulation of cytochrome C expression through translation enhancer and repressor function respectively.^46^ HuR was found to stabilize COQ7 enzyme and thereby regulates mitochondrial function through CoQ biosynthesis.^47^ Increased translation efficiency mediated by HuR on the OPA1 regulation promoted mitochondrial respiratory activity and the findings emphasis the role of HuR in mitochondrial physiology.^48^ Previously findings from our group report on the role of HuR in the up‐regulation of autophagy and suppression of apoptosis under hypoxic stress.^30^ Here, we show that silencing HuR expression abolished hypoxia‐regulated mitophagy through the decreased formation of mitophagosome and mitolysosome. Thus, HuR might regulate hypoxia‐induced cell stress in renal tubular cells through enhanced mitophagy.

Mitophagy is regulated by PINK1/PARKIN and BNIP3/BNIP3L dependent pathways. PINK1, PTEN‐induced Kinase 1 acts as a sensor for depolarized mitochondria and recruits cytosolic PARKIN, RBR E3 ubiquitin‐protein ligase. The recruited PARKIN in the mitochondria ubiquitinates outer mitochondrial membrane proteins and directs for autophagic degradation.^49^ The other pathway involves BNIP3L/NIX and BNIP3, which belong to the family of BH3‐only Bcl‐2 pro‐apoptotic proteins. Under mitophagy activation, BNIP3 and BNIP3L translocate to the dysfunctional mitochondria and interact with LC3/GABARAP proteins at their N‐terminal site to promote mitochondrial degradation.^50^ In this study, we show evidence that HuR regulates PARKIN and BNIP3L protein expressions under hypoxic conditions. Increased HuR binding and RNA stabilization of *PARKIN* and *BNIP3L* mRNA promoted its expression under hypoxia. The importance of PARK2‐mediated mitophagy induction in sepsis‐induced acute kidney injury (SI‐AKI) has been demonstrated earlier.^51^ PINK1 and PARKIN knockdown studies in CI‐AKI showed the protective role of PINK1‐PARKIN‐mediated mitophagy in reducing oxidative stress and NLRP3 inflammasome‐mediated apoptosis.^52^ Protective role of PINK1/PARKIN‐mediated mitophagy in cisplatin‐induced AKI, renal ischemia‐reperfusion injury and renal epithelial cell injury has been reported in PINK1 and PARKIN knockout (KO) models.^53^, ^54^, ^55^ BNIP3/BNIP3L family of proteins are regarded as mitochondrial stress sensors.^56^ BNIP3/NIX‐mediated mitophagy and ROS removal regulated NK cell survival and memory.^57^ Yuan and colleagues in 2017 demonstrated the neuroprotective roles of BNIP3L against ischaemic brain injury through the regulation of mitophagy.^58^ The present findings highlight that HuR regulates mitophagy in renal tubular cells through post‐transcriptional mRNA stabilization of *PARKIN/BNIP3L*.

## 5 CONCLUSION

In conclusion, hypoxia induces mitophagy through mitophagosome and mitolysosome formation in renal tubular cells. The findings show that RNA‐binding protein‐HuR regulates mitophagy through RNA stabilization of *PARKIN* and *BNIP3L* expressions.

## CONFLICT OF INTEREST

The authors confirm that there are no conflicts of interest.

## AUTHOR CONTRIBUTIONS

**Shao‐Hua Yu:** Conceptualization (lead); Data curation (equal); Funding acquisition (lead); Investigation (lead); Methodology (lead); Resources (equal); Supervision (equal); Validation (equal); Writing‐original draft (lead); Writing‐review & editing (equal). **Kalaiselvi Palanisamy:** Conceptualization (lead); Data curation (equal); Formal analysis (equal); Funding acquisition (supporting); Methodology (lead); Validation (equal); Visualization (equal); Writing‐original draft (lead); Writing‐review & editing (equal). **Kuo‐Ting Sun :** Conceptualization (lead); Funding acquisition (lead); Investigation (lead); Methodology (equal); Resources (lead); Supervision (lead); Visualization (lead); Writing‐review & editing (equal). **Xin Li:** Data curation (supporting); Formal analysis (equal); Methodology (equal); Software (equal); Validation (equal); Visualization (equal). **Yao‐Ming Wang :** Investigation (equal); Project administration (equal); Resources (equal); Supervision (equal); Visualization (equal). **Feng‐Yen Lin:** Formal analysis (equal); Investigation (equal); Project administration (equal); Visualization (equal). **Kuen‐Bao Chen:** Formal analysis (equal); Project administration (equal); Resources (supporting); Visualization (equal). **I‐Kuan Wang:** Conceptualization (equal); Funding acquisition (lead); Investigation (equal); Project administration (equal); Resources (lead); Supervision (lead). **Tung‐Min Yu:** Conceptualization (equal); Funding acquisition (lead); Project administration (lead); Resources (lead); Supervision (lead); Visualization (equal). **Chi yuan Li:** Conceptualization (equal); Funding acquisition (lead); Investigation (lead); Resources (lead); Supervision (lead); Writing‐review & editing (lead).

## Data Availability

The data used to support the findings of this study are available from the corresponding author upon reasonable request.
